# Letter to the Editor^☆^

**DOI:** 10.1016/j.bjorl.2015.08.002

**Published:** 2015-09-07

**Authors:** Satvinder Singh Bakshi

**Affiliations:** Department of ENT and Head & Neck Surgery, Mahatma Gandhi Medical College and Research Institute, Pillaiyarkuppam, India

Dear Editor,

I am writing to you in reference to a very thought provoking article titled ‘Derivation of a clinical decision rule for predictive factors for the development of pharyngocutaneous fistula postlaryngectomy’ by Cecatto et al.,^1^ in your esteemed journal. The article is well written; however, there are some more factors in the etiology of pharyngocutaneous fistula that the authors have not analyzed and that could affect the outcome, which I would like to highlight.

The etiology of pharyngocutaneous fistula is multifactorial, which has been proven by multiple studies^2^ and reviews.^3^ There are several additional proven significant factors in the etiology of pharyngocutaneous fistula, which have not been analyzed in this study, such as intra-operative blood transfusion,^3^ surgical duration,^3^ and presence of hypothyroidism.^4^ The inclusion of these may change the multivariate analysis and ultimately the conclusion.

The study is very novel and the development of clinical decision rule for pharyngocutaneous fistula is quite commendable. But there is concern regarding the sampling of the patients; for instance, the number of patients receiving pre-operative chemoradiotherapy (CRT), just 15 (8.8%), which is too small for deriving a conclusion, since CRT has proven to be one of the most significant factors in development of pharyngocutaneous fistula.^3^, ^4^ The study also includes few patients with stage I disease, who underwent laryngectomy. This does not adhere to the current guideline, and therefore these patients could have been excluded.

The stratification of patients into risk groups is a good idea for economic use of resources, but if we study the stratification carefully, the sensitivity for high-risk groups is just 48%, which is low for such an important complication and casts a doubt on its clinical application.

## Conflicts of interest

The author declares no conflicts of interest.
